# Angular reproduction numbers improve estimates of transmissibility when disease generation times are misspecified or time-varying

**DOI:** 10.1098/rspb.2023.1664

**Published:** 2023-09-27

**Authors:** Kris V. Parag, Benjamin J. Cowling, Ben C. Lambert

**Affiliations:** ^1^ MRC Centre for Global Infectious Disease Analysis, Imperial College London, London, UK; ^2^ NIHR Health Protection Research Unit in Behavioural Science and Evaluation, University of Bristol, Bristol, UK; ^3^ WHO Collaborating Centre for Infectious Disease Epidemiology and Control, School of Public Health, The University of Hong Kong, Hong Kong Hong Kong; ^4^ Department of Mathematics, College of Engineering, Mathematics and Physical Sciences, University of Exeter, Exeter, UK; ^5^ Department of Statistics, University of Oxford, Oxford, UK

**Keywords:** infectious diseases, epidemic models, reproduction numbers, generation times, growth rates, transmission dynamics

## Abstract

We introduce the *angular reproduction number Ω*, which measures time-varying changes in epidemic transmissibility resulting from variations in both the effective reproduction number *R*, and generation time distribution *w*. Predominant approaches for tracking pathogen spread infer either *R* or the epidemic growth rate *r*. However, *R* is biased by mismatches between the assumed and true *w*, while *r* is difficult to interpret in terms of the individual-level branching process underpinning transmission. *R* and *r* may also disagree on the relative transmissibility of epidemics or variants (i.e. *r_A_* > *r_B_* does not imply *R_A_* > *R_B_* for variants *A* and *B*). We find that *Ω* responds meaningfully to mismatches and time-variations in *w* while mostly maintaining the interpretability of *R*. We prove that *Ω* > 1 implies *R* > 1 and that *Ω* agrees with *r* on the relative transmissibility of pathogens. Estimating *Ω* is no more difficult than inferring *R*, uses existing software, and requires no generation time measurements. These advantages come at the expense of selecting one free parameter. We propose *Ω* as complementary statistic to *R* and *r* that improves transmissibility estimates when *w* is misspecified or time-varying and better reflects the impact of interventions, when those interventions concurrently change *R* and *w* or alter the relative risk of co-circulating pathogens.

## 1. Introduction

Estimating the rate of spread or transmissibility of an infectious disease is a fundamental and ongoing challenge in epidemiology [1]. Identifying salient changes in pathogen transmissibility can contribute important information to policymaking, providing useful warnings of resurgent epidemics, assessments of the efficacy of interventions and signals about the emergence of new variants of concern [1–3]. The effective or instantaneous reproduction number, *R*, and time-varying growth rate, *r*, are commonly used to characterize pathogen transmissibility. The former statistic is an estimate of the average number of new infections per active (circulating) past infection, while the latter describes the exponential rate of new infection accumulation [4].

**Figure 1.**
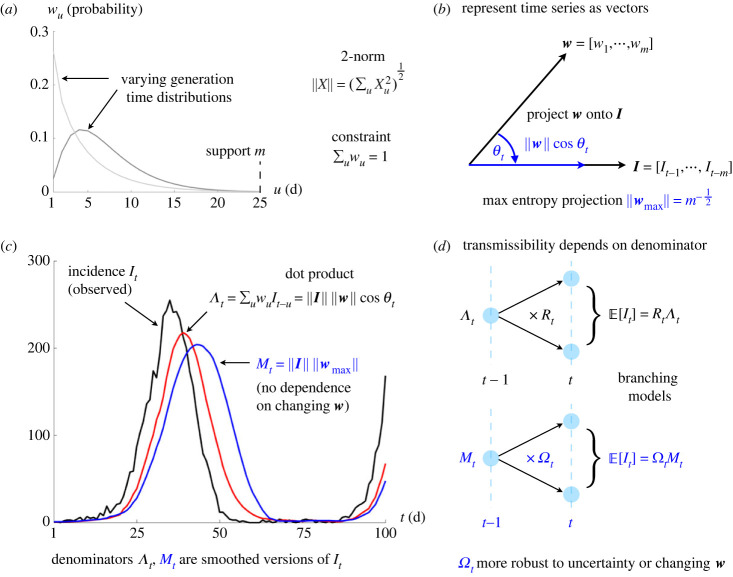
Definitions of transmissibility metrics. Panel (*a*) plots generation time distributions that define how past infections cause later ones via the probabilities or weights *w* with support *m*. This involves convolving these weights with past infection incidence *I*. We show in (*b*) that if we represent *w* and *I* as vectors then the convolution is equivalent to a projection of *w* onto the vector of *I*. Panels (*c,d*) illustrate that standard reproduction numbers *R* implicitly apply this projection to compute the denominator *Λ*. This projection and hence *Λ* is sensitive to the *w*, meaning that if the distribution switches between the two from (*a*), our estimates of *R* become biased (often changes in generation time are difficult to measure). Our new metric *Ω* maximizes the projection from (*b*) to reduce sensitivity (practically this involves a window based on *m*) leading to a new denominator *M* in (*c*). This maintains the branching process interpretation of the epidemic in (*d*), while improving transmissibility estimate robustness.

Although *R* and *r* are important and popular means of tracking the dynamics of epidemics, they suffer from key limitations that diminish their fidelity and interpretability. Specifically, the meaningfulness of *R* depends on our ability to measure the generation time distribution of the infection under study, *w*. This distribution captures the inter-event times among primary and secondary infections [5] and is convolved with the past infections to define *Λ*, the time-varying total infectiousness of the disease. The total infectiousness serves as the denominator when inferring *R*, which is the ratio of new infections to *Λ*. We illustrate all key notation in figure 1. However, infection times and hence *w* are difficult to measure, requiring detailed transmission chain data from contact tracing or transmission studies [6]. Even if these data are available, the estimated *w* (and hence *Λ*) depends on how inter-event times are sampled or interpreted (e.g. there are forward, backward, intrinsic and realized generation intervals) [7,8].

Workarounds, such as approximating *w* by the serial interval distribution [9], which describes inter-event times between the onset of symptoms, or inferring *w* from this distribution [10], do exist but suffer from related problems [6]. Consequently, *w* and *Λ* can often be misspecified, biasing *R* and likely misrepresenting the true branching process dynamics of epidemics. While *r* is more robust to *w* misspecification (it only depends on the log gradient of the smoothed infection time series) [4], it lacks the individual-level informativeness and interpretation of *R*. Given estimates of *r*, it is unclear how to derive the proportion of new infections that need to be suppressed and herd immunity thresholds (both related to 1 − *R*^−1^) or the probability of epidemic elimination and establishment (both linked to *R^−N^* for *N* infections) [11–13]. The only known means of attaining such information converts *r* into *R* using estimates of *w* [14].

Difficulties in accurately inferring generation times therefore cause practical bottlenecks that constrain our ability to measure pathogen transmissibility. These problems are worsened as recent studies have empirically found that generation times also vary substantially with time (i.e. *w* is non-stationary) [15]. These variations may correspond to different epidemic phases [16], emerging variants of concern [17] and coincide with the implementation of interventions [18]. These are precisely the situations in which we also want to infer *R*. However, concurrent changes in *R* and *w* are rarely identifiable, and *r* inextricably groups the effects of *w* and *R* on transmissibility. While high quality, longitudinal contact tracing data [19] can potentially resolve these identifiability issues, this is an expensive and logistically hard solution. Here we propose another means of alleviating the above problems and complementing the insights provided by *R* and *r*—the *angular reproduction number*, *Ω*.

The angular reproduction number defines transmissibility as a ratio of new infections to *M*, the root mean square number of past infections over a user-defined window *δ*. Because it replaces *Λ* with *M*, a quantity that does not require knowledge of generation times, *Ω* is more robust to the problems of inferring *w*. We demonstrate that *Ω* is able to measure the overall changes in transmissibility caused by fluctuations in both *R* and *w*. Moreover, we prove that *Ω* has similar threshold properties to *R*, maintains much of its individual-level interpretation and is a useful metric for communicating transmissibility. This last point follows as we only need to quote *Ω* and the known window *δ* to generalize our estimates of transmissibility to different settings. By contrast, the meaningfulness of *R* is contingent on the unknown or uncertain *w*. Downstream studies sometimes use *R* outside of its generation time context [20], while dashboards aiming at situational awareness commonly quote *R* without *w*, introducing biases and interpretability problems into how disease spread is communicated [21].

Additionally, we demonstrate how *r* and *R* can easily disagree on relative transmissibility, both across time and for co-circulating variants. Unmeasured changes in *w* over time can cause *R* and *r* to vary in opposite directions (one signals an increase in transmissibility and the other a decrease). Similarly, co-circulating pathogens with different but stationary and known *w* may possess contradictory *R* and *r* value rankings, i.e. for variants *A* and *B*, *r_A_* > *r_B_* does not imply *R_A_* > *R_B_*. These issues are amplified when interventions (which can change *w*, *R* or both [18]) occur, obscuring notions of the relative risk of spread. However, we find *r_A_* > *r_B_* guarantees *Ω_A_* > *Ω_B_* and that *Ω* is consistent with *r* across time even when *w* changes.

Last, while we may also convert *r* into threshold statistics about 1 by using a free parameter together with a transformation from [14], we show that *Ω* is more robust to choices of its free parameter than those statistics, which implicitly make stronger assumptions (electronic supplementary material information). These robustness and consistency properties of *Ω* reinforce its usefulness for tracking and comparing outbreak spread and emerge from its maximum entropy approach to managing uncertain generation time distributions. We propose *Ω* as a complementary statistic that can be integrated with *R* and *r* to present a more comprehensive perspective on epidemic transmissibility, especially when *w* is poorly specified or varying with time.

## 2. Results

### (a) Angular reproduction numbers

The epidemic *renewal model* [22] provides a general and flexible representation of disease transmission. It defines how the incidence of new infections at time *t*, denoted *I_t_*, depends on the effective or *instantaneous reproduction number*, *R_t_*, and the past incident time series of infections, $I_{1}^{t-1}:=\{I_{1},I_{2},\ldots I_{t-1}\}$. This results in the conditional moment relationship in equation (2.1) [9]. Generally, we use $X_{a}^{b}$ to denote the time series {*X_a_*, *X_a_*_+1_, … , *X_b−_*_1_, *X_b_*} and **E**[*X*|*Y*] for the expectation of *X* over possible epidemic trajectories given known variables *Y*. Where obvious, and for convenience, we sometimes drop *Y* in **E**[*X*|*Y*], writing **E**[*X*].

2.1 $$E[I_{t}|\;I_{1}^{t-1},w_{1}^{m}]=R_{t}\Lambda_{t},\;\Lambda_{t}=\overset{m}{\underset{u=1\;}{\sum }}w_{u}I_{t-u\;}.\;$$

In this model, Λ*_t_* is known as the *total infectiousness* and summarizes the weighted influence of past infections. The set of weights *w_u_* for all *u* defines the *generation time distribution* of the infectious disease with $\sum_{u=1}^{m}w_{u}=1$, and *m* as the support of this distribution, which we assume to be practically finite [14]. When the time series is shorter than *m* we truncate and renormalize the *w_u_*. Commonly, the stochasticity around the expectation *R_t_*Λ*_t_* is modelled using either Poisson or negative binomial count distributions [1,12].

Although equation (2.1) has successfully been applied to model many diseases including COVID-19, Ebola virus disease, pandemic influenza and measles, among others, it has one major flaw—it assumes that the generation time distribution is fixed or stationary and known [9]. If this assumption holds (we ignore surveillance biases [9,23] until the Discussion), equation (2.1) allows epidemic transmissibility to be summarized by fluctuations of the time-varying *R_t_* parameters. This follows because the sign of *R_t_* − 1 determines if *I_t_* will increase or decline relative to the total infectiousness Λ*_t_*. This reproduction number can be linked to the *instantaneous epidemic growth rate*, *r_t_*, using the moment generating function of the generation time distribution [14].

Consequently, from *R_t_*, we obtain temporal information about the rate of pathogen spread and its mechanism, i.e. we learn how many new infections we can expect per circulating infection because $R_{t}=E[I_{t}]\Lambda_{t}^{-1}$. As *R_t_* is a threshold parameter, we know that we must block at least a fraction $1-R_{t}^{-1}$ of new infections to suppress epidemic growth (*R_t_* = 1 signifies that *r_t_* = 0). The time scale over which this suppression is achievable [14] and our ability to detect these changes in *R_t_* [24] in the first place, however, are determined by the generation times.

Recent works emphasize that the assumption of a known or fixed generation time distribution is often untenable, with appreciable fluctuations caused by interventions [15,18] and emerging pathogenic variants [17] or occurring as the epidemic progresses through various stages of its lifetime [5]. Substantial biases in *R_t_* can result (because its denominator Λ*_t_* is incorrectly specified [4]), which even impede optimal Bayesian inference algorithms [25]. As *R_t_* is the predominant metric of transmissibility, contributing key evidence towards infectious disease policymaking [1], this may potentially obscure situational awareness or misinform intervention planning. While improved and intensive contact tracing can provide updated generation time information, this is usually difficult and expensive. We propose a robust alternative.

We redefine Λ*_t_* by recognizing it as a dot product between the vectors of generation time probabilities $w:=w_{1}^{m}$ and the past incidence $I:=I_{t-m}^{t-1}$ over the support of the generation time distribution, *m*. This gives the left equality of equation (2.2) with the Euclidian norm of ***X*** as $\|X\|:={\left(\sum_{u=1}^{m}X_{u}^{2}\right)}^{1/2}$ and *θ_t_* as the time-varying angle between ***w*** and ***I***. This equality holds for non-stationary generation times, i.e. both ***w*** and ***I*** can have elements that change over time. We illustrate this notation and elements of the subsequent derivation in figure 1. Equation (2.2) implies that the count of new infections (for any *R_t_*) is maximized when *θ_t_* is minimized, i.e. when the temporal profile of past infections matches the shape of the generation time distribution.

2.2 $$\Lambda_{t}=\|w\|\|I\|\cos\theta_{t},\;E[I_{t}]=\left(\frac{\left\|w\right\|}{\left\|w_{\max}\right\|}R_{t}\cos\theta_{t}\right)M_{t}.\;$$

We can compute the root mean square of the incidence across the support of the generation time distribution as $M_{t}:=(1/\sqrt{m})\|I\|$. Under the constraint that $\sum_{u=1}^{m}w_{u}=1$ (if *t* − 1 < *m*, we truncate this distribution to sum to 1—this is an edge effect of the epidemic) then the maximum possible value of the generation time norm is $\|w_{\max}\|=1/\sqrt{m}$. This is achieved by the maximum entropy generation time distribution of ***w***, which is uniform (has *m* entries of 1/*m*).

Combining these definitions with equation (2.1), we derive the second expression in equation (2.2) for the expected number of new infections at time *t*. This may seem an unnecessarily complicated manipulation of the standard renewal model, but it admits a novel and important insight—we can separate the influences of the reproduction numbers and the generation time distribution (together with its changes) on epidemic transmissibility. These multiply *M_t_*, which defines a new denominator—the root mean square number of past infections (this is also the average signal power of the past infection time series)—that replaces the total infectiousness Λ*_t_*.

Consequently, we define a new metric in equation (2.3), the *angular reproduction number* Ω*_t_*, which multiplies *R_t_* by the scaled projection of the generation time distribution, $(\|w\|/\|w_{\max}\|)\cos\theta_{t}$, onto ***I***, the past incidence vector (figure 1). This means that Ω*_t_* is a time-varying ratio between the expected infection incidence and the past root mean square incidence *M_t_*. We use the term reproduction number for Ω*_t_* due to its relation to *R_t_*, the similarity of equation (2.1) and equation (2.3) and because of its threshold properties, which we explore in the next section.

2.3 $$\Omega_{t}:=\frac{\left\|w\right\|}{\left\|w_{\max}\right\|}R_{t}\cos\theta_{t}\Rightarrow \Omega_{t}=E[I_{t}]M_{t}^{-1}.\;$$

This metric captures all possible variations that impact the ability of the epidemic to transmit. It responds to both changes in *R_t_* and the generation time distribution. The latter would scale ‖w‖ and rotate *θ_t_*, which is why we term this angular. The benefit of compactly describing both types of transmissibility changes does come with a trade-off in interpretability as it may be harder to intuit the meaning behind $E[I_{t}]=\Omega_{t}M_{t}$ than the more usual $E[I_{t}]=R_{t}\Lambda_{t}$.

We argue that this is not the case practically because Λ*_t_* is frequently misspecified [15,26], obscuring the meaning of *R_t_*. In contrast, *M_t_* does not depend on generation time assumptions (beyond characterizing its support *m*). We remove structural uncertainty induced by the often unknown *w_u_* because *M_t_* is a maximum entropy version of Λ*_t_*, i.e. $M_{t}=\underset{\|w\|\cos\theta_{t}}{\max}\Lambda_{t}=\;\|w_{\max}\|\|I\|$ subject to $\sum_{u=1}^{m}w_{u}=1$. We also find that *M_t_* = Λ*_t_* and hence Ω*_t_* = *R_t_*, when the past incidence is flat (as then *w_u_* has no effect). This defines the important and universal equilibrium condition Ω*_t_* = *R_t_* = 1. There is further convergence for branching process models [27] with timesteps at its fixed generation time, as then trivially *w*_1_ = 1.

### (b) Relationship to popular transmissibility metrics

Having defined the angular reproduction number above, we explore its properties and show why it is an interesting and viable measure of transmissibility. We examine an exponentially growing epidemic with incidence *I_t_* = *I*_0_*e^rt^* and constant growth rate *r*. This model matches the dynamics of fundamental *compartmental models* such as the SIR and SEIR (in the limit of an excess of susceptible individuals) and admits the known relation *gr* = (*R* − 1) [28], with *g* as the mean generation time. We assume growth occurs over some period of *δ* and compute Ω*_t_* as the ratio $E[I_{t}]M_{t}^{-1}$ from equation (2.3). Since this model is deterministic $E[I_{t}]=I_{t}=I_{0}e^{rt}$.

We evaluate *M_t_* from its definition above as $\left(1/\sqrt{m})\|I\right\|$ with *δ* = *m* and using the continuous-time expression for $\|I\|={\left(\int_{t-\delta }^{t}I_{s}^{2}\;ds\right)}^{1/2}$. This yields $M_{t}={\left(\delta^{-1}\int_{t-\delta }^{t}I_{s}^{2}\;ds\right)}^{1/2}$ with $I_{s}^{2}=I_{0}^{2}e^{2rs}$ from the exponential incidence equation and evaluates to $\sqrt{2\delta rI_{0}(e^{2rt}-e^{2r(t-\delta )})}$. Substituting this into $\Omega_{t}=(I_{0}e^{rt})M_{t}^{-1}$ results in the left relation in equation (2.4).

2.4 $$\Omega_{t}^{2}=\frac{2\delta r}{1-e^{-2\delta r\;}}\geq 1,\;\Omega_{t}^{2}=\frac{2\delta g^{-1}(R-1)}{1-e^{-2\delta g^{-1}(R-1)}}.\;$$

Several important points follow. First, as *x* ≥ 1 − *e*^−^*^x^* for every *x* ≥ 0, then Ω*_t_* − 1 and *r* are positive too (an analogous argument proves the negative case). Second, we substitute for *r* using the compartmental *R*–*r* relationship *gr* = (*R* − 1) to get the right-side relation of equation (2.4). Applying L’Hôpital's rule we find $\underset{R\to 1}{\lim}\Omega_{t}=1$. We hence confirm the threshold behaviour of Ω*_t_*, i.e. the signs of Ω*_t_* − 1 and *R_t_* − 1 are always consistent (for all values of *δ* > 0).

**Figure 2.**
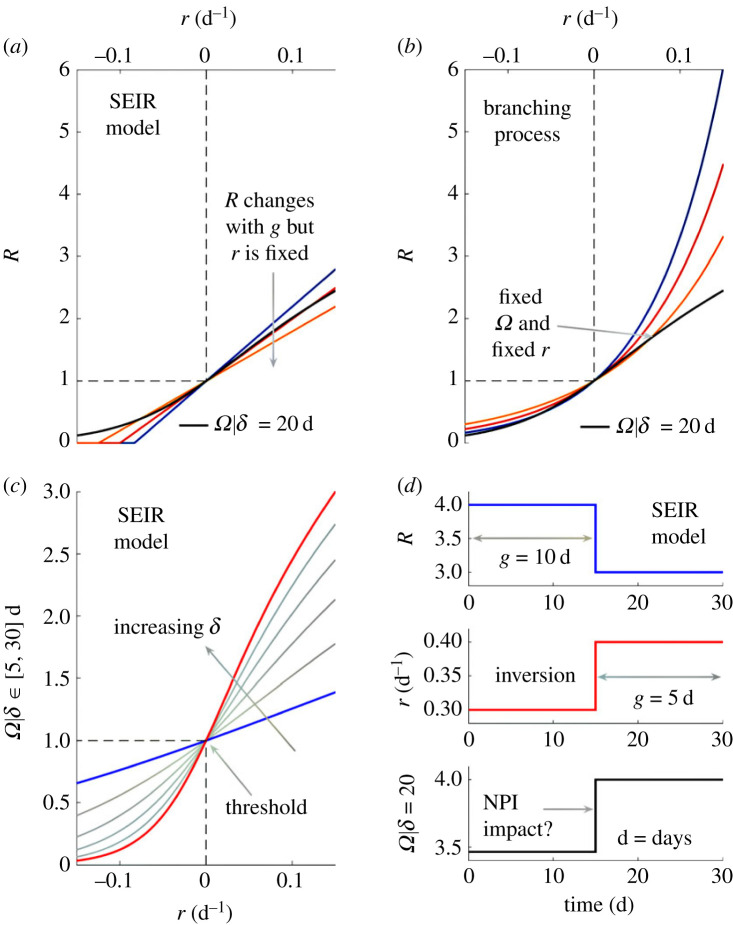
Relationships among transmissibility metrics. Panels (*a*) and (*b*) show how growth rates (*r*) and reproduction numbers (*R*) have diverse functional relationships (see [14,29]) for SEIR models with an excess of susceptible individuals and branching processes. Coloured lines indicate *R* at different mean generation times (*g*). Black lines highlight a single functional relationship between angular reproduction numbers *Ω* and *r* at all *g*, using a window *δ* of 20 d. Panel (*c*) shows that while *Ω* varies with choice of *δ* (increasing from blue to red and computed from equation (2.4)), we have a bijective relationship with *r*. Panel (*d*) indicates that *R* and *r* can signify inverted changes, e.g. an NPI reducing *R* and *g* may increase *r*, raising questions about impact (see [15,18]). Here, *Ω* converts *r* into a consistent transmissibility metric (also from equation (2.4)).

Third, we see that constant growth rates imply constant angular reproduction numbers. The converse is also true, and we may input time-varying growth rates, *r_t_*, into equation (2.4) to estimate Ω*_t_*. These properties hold for any *δ*, which is now a piecewise-constant time window. We plot the ramifications of equation (2.4) in figure 2. Further, in table 1, we summarize how Ω*_t_* relates to predominant *R_t_* and *r_t_* metrics. We explore some properties in this table in later sections (in addition to reinforcing our analyses with stochastic models) and demonstrate that relationships among *r_t_*, *R_t_* and Ω*_t_* have important consequences when comparing outbreaks subject to interventions and variations in generation times.

**Table 1.** Summary of transmissibility metrics. We list important relationships among the instantaneous growth rate (*r*), the instantaneous or effective reproduction number (*R*) and the angular reproduction number (*Ω*) and assess their value as measures of transmissibility.

metric property	growth *r*	effective *R*	angular *Ω*
definition of transmissibility	$r_{t}:=\frac{d\log E[I_{t}]}{dt}$	$R_{t}:=\frac{E[I_{t}]}{\Lambda_{t}}$	$\Omega_{t}:=\frac{E[I_{t}]}{M_{t}}$
pathogen spread threshold	*r_t_* > 0	*R_t_* > 1	Ω*_t_* > 1
biased by generation time ***w*** assumed, given curve $I_{1}^{t}$	insensitive to the assumed ***w***	biased when ***w*** is misspecified	signals change in w and *R_t_*
ranking risk of outbreaks or variants by spreading rate	*r_A_* > *r_B_* ⇒ variant *A* spreads faster	*r*_*A*_ > *r*_*B*_ ⇏ *R*_*A*_ > *R*_*B*_ (inconsistent)	*r_A_* > *r_B_* ⇒ Ω*_A_* > Ω*_B_* (consistent)
short-term predictive power	negligible differences among metrics in prediction quality		
non-dimensional metric	no, inverse time	yes, both have no units, scalable	
individual-level interpretability	not obvious	new infections per circulating ones	
computability if ***w*** unknown	yes (smooth $I_{1}^{t}$)	not possible	yes, for any *δ*

Note that we may also invert the relationship in equation (2.4) to estimate *r_t_* from Ω*_t_* (see Methods for details). This involves solving equation (2.5), where *W_k_*(*x*) is the Lambert W function with index *k* ∈ [0, − 1] (this range results from the indicator 1(*y*)) [30].

2.5 $$\frac{d\log I_{t}}{dt}=r_{t}=2\delta^{-1}(\Omega_{t}^{2}+W_{-1(\Omega <1)}(-\Omega_{t}^{2}e^{-\Omega_{t}^{2}})).\;$$

A central implication of equation (2.4) and equation (2.5) is that we can infer angular reproduction numbers directly from growth rates or vice versa, without requiring knowledge of the generation times.

We further comment on connections between angular and effective reproduction numbers using a deterministic *branching process* model, which is also foundational in epidemiology. We again focus on growth, which is geometric as this is a discrete-time process with time steps scaled in multiples of the mean generation time *g*. Here, incidence is *I_t_* = *R^t^* and $\Omega_{t}=E[I_{t}]M_{t}^{-1}=R^{t}{\left(\delta^{-1}\sum_{s=t-\delta }^{t-1}R^{2s}\right)}^{-1/2}$, with window *δ* in units of *g*. If *δ* = 1, we recover Ω*_t_* = *R*. If *R* = 1, then Ω*_t_* = *R* for all *δ*. For growing epidemics, as *δ* increases, Ω*_t_* > *R* because we reference present incidence to smaller past infections (or denominators). The opposite occurs if the epidemic declines. This may seem undesirable, but we argue that Ω*_t_* improves overall practical transmissibility measurement because *g* will likely be misspecified or vary with time.

Any *g* mismatches bias *R*, limiting its interpretation, meaningfulness and making comparisons among outbreaks or pathogenic variants difficult, because we cannot be certain that our denominators correspond. This is particularly problematic when estimates of *R* obtained from a modelling study are incorporated as parameters into downstream studies without accounting for the generation time context on which those estimates depend. However, by additionally communicating *Ω* and *δ*, we are sure that denominators match and that we properly include the influences of any *g* mismatches. Choosing *δ* is also no worse (and more explicit) than equivalent window assumptions made when inferring *R* and *r* [4,29]. In the electronic supplementary material information, we perform analyses of window choices for *Ω* and other threshold metrics.

Last, we illustrate how Ω*_t_* relates to other key indicators of epidemic dynamics such as herd immunity and elimination probabilities. As our derivation replaces equation (2.1) with $E[I_{t}|\;I_{1}^{t-1},\delta ]=\Omega_{t}M_{t}$ for the same observed incidence, these indicators are also readily obtained. Assuming Poisson noise, the elimination probability $\prod_{s=t}^{\infty }P[I_{t}=0|\;I_{1}^{t-1},R_{1}^{t-1}]=e^{-\sum_{s=t}^{\infty }\Lambda_{t}R_{t}}$ is replaced by $e^{-\sum_{s=t}^{\infty }M_{t}\Omega_{t}}$, and has analogous properties [31]. Herd immunity, which traditionally occurs when a fraction 1 − *R*^−1^ of the population is immune is approximated by 1 − Ω^−1^ (since both metrics possess the same threshold behaviour) [11]. In a subsequent section, we demonstrate that one-step-ahead incidence predictions from both approaches are also comparable.

### (c) Responding to variations in generation time distributions

We demonstrate the practical benefits of Ω*_t_* using simulated epidemics with non-stationary or time-varying generation time distributions. Such changes lead to misspecification of Λ*_t_* in equation (2.1), making estimates of the effective reproduction number *R_t_*, denoted ${\hat{R}}_{t}$, a poor reflection of the true underlying *R_t_*. By contrast, variations in the estimated ${\hat{\Omega }}_{t}$ are a feature (see equation (2.3)) and not a bug (for some chosen *δ* we control *M_t_*, which is not misspecified). We simulate epidemics with Ebola virus or COVID-19 generation times from [32,33] using renewal models with Poisson noise [9]. We estimate both the time-varying *R_t_* and Ω*_t_* using *EpiFilter* [25], which applies Bayesian algorithms that minimize mean square estimation error.

Inferring Ω*_t_* from incident infections, $I_{1}^{t}$, requires only that we replace the input Λ*_t_* with *M_t_* in the estimation function and that we choose a window *δ* for computing *M_t_*. We provide software for general estimation of Ω*_t_* and code for reproducing this and all other analyses in this paper at https://github.com/kpzoo/Omega. We heuristically set *δ* ≈ 2*g*_0_ as our window with *g*_0_ as the original mean generation time of each disease from [32,33]. We find (numerically) that this *δ* ensures $\sum_{u=0}^{\delta }w_{u}\geq 0.86$ over many possible gamma distributed generation times, i.e. it is long enough to cover most of the likely probability mass of unknown changes to the generation time distributions, which cause time-varying means *g_t_*. In general, we find that an overly small *δ* tends to neglect important dynamics, while too large a *δ* induces edge effects. The Methods and electronic supplementary material information provide for more information on choosing *δ*.

**Figure 3.**
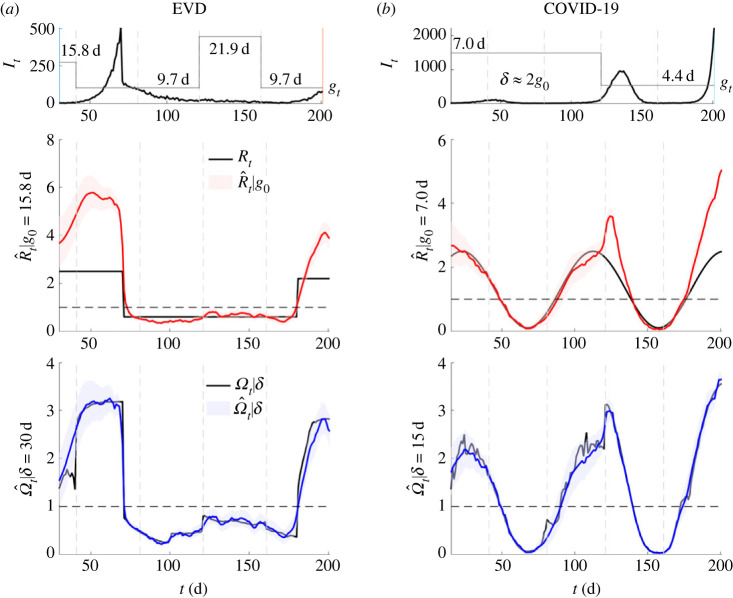
Estimating transmissibility under temporal variations in generation times. We simulate epidemic incidence curves (black) using generation time distributions of (*a*) Ebola virus disease (EVD) [32] and (*b*) COVID-19 [33]. The means of these distributions (*g*) vary over time (grey piecewise, starting from original mean *g*_0_), but we fix their variance at their original values. We find substantial bias in *R* estimated from the initial EVD and COVID-19 generation times (red with 95% credible intervals, true value in black). These estimates try to compensate for generation time mismatches and changes in an uncontrolled manner that obscures interpretation. However, *Ω* responds as we expect (blue with 95% credible intervals, window *δ*, true value in black) and we infer change-points due to both *R* and *g* fluctuations (subject to bounds induced by noise, i.e. at low incidence inference is more difficult [24]). Our estimates derive from EpiFilter [25] with default settings and we truncate time series to start from *δ* to remove any edge effects. Vertical dashed lines highlight times at which we change *g* or keep it fixed. When it is fixed, *Ω* infers no spurious changes.

Our results are plotted in figure 3. We show that ${\hat{\Omega }}_{t}$ responds as expected to both changes in the true *R_t_* and $w_{1}^{m}$, subject to the limits on what can be inferred [24]. In figure 3, we achieve changes in $w_{1}^{m}$ by altering the mean generation time *g_t_* by ratios that are similar in size to those reported from empirical data [15]. By contrast, we observe that ${\hat{R}}_{t}$ provides incorrect and overconfident transmissibility estimates, which emerge because its temporal fluctuations also have to encode structural differences due to the misspecification of $w_{1}^{m}$. These can strongly mislead our interpretation and understanding of the risk posed by a pathogen.

We can derive alternative threshold statistics that relate to *r_t_* and do not explicitly depend on the generation time by applying monotonic transformations from [14]. In theory, these should have comparable behaviour around the critical point of 1 to both *R_t_* and Ω*_t_*. We investigate these statistics in the electronic supplementary material information, computing them across the simulations of figure 3. We find that they require stronger assumptions than Ω*_t_* (i.e. they fix distributional formulae for generation times), possess at least as many free parameters as Ω*_t_* and are less robust to changes in those parameters (often strongly over-estimating transmissibility) than Ω*_t_* is to fluctuations in *δ*. This confirms that angular reproduction numbers can complement standard metrics, improving transmissibility estimates when generation times are changing, or unknown and forming part of a more comprehensive suite of outbreak diagnostics.

### (d) Ranking epidemics or variants by transmissibility

Misspecification of generation time distributions, and corresponding misestimation of *R* as in figure 3, also plays a crucial role when assessing the relative transmissibility of pathogens, variants of concern or even outbreaks (where we may want to contrast the spread of contagion among key demographic or spatial groups). As shown in figure 2, these variations can mean that increases in the growth rate *r_t_* actually signify decreases in the effective reproduction number *R_t_* or that a pathogen with a larger *r_t_* can have a smaller *R_t_*. Here, we illustrate that these issues can persist even if the generation time distributions of pathogens are correctly specified and remain static, obscuring our understanding of relative transmission risk.

**Figure 4.**
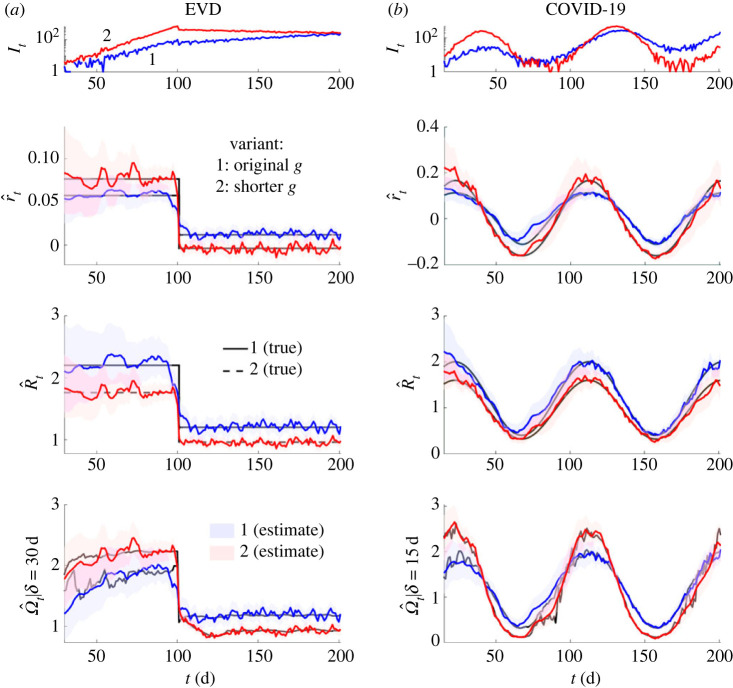
Comparing transmissibility across outbreaks, variants or even diseases. We simulate epidemics of variant 1 in blue (with estimates of metrics also in blue) under standard generation time distributions of (*a*) Ebola virus disease (EVD) [32] and (*b*) COVID-19 [33]. In red (with estimates also in red), we overlay simulations in which the generation time of these diseases is 40% and 50% shorter (than the blue epidemics), which may indicate a new co-circulating variant 2 or another epidemic with different properties (e.g. in a higher risk group). We demonstrate (for the first time to our knowledge) that changes in *R* due to an intervention (or release of one) may invert the relative growth rates (*r*) of the epidemics (see electronic supplementary material information for mathematical intuition for this inversion). The mismatches in the *R–r* rankings alter perceptions of relative risk, making transmissibility comparisons difficult. However, *Ω* classifies the risk of these epidemics in line with their realized growth rates, while still offering the individual-level interpretability of a reproduction number. True values are in black and all estimates (with 95% credible intervals) are outputs from EpiFilter [25] with default settings. We truncate the time series to start from *δ* to remove any edge effects.

In figure 4, we simulate epidemics under two hypothetical variants of two pathogens. We use EVD and COVID-19 generation time distributions from [32,33] to define our respective base variants. For both pathogens, we specify the other variant by reducing the mean generation of each base but fixing the variance of the generation times. Reductions of this type are plausible and have been measured for COVID-19 variants [17]. All $w_{1}^{m}$ distributions are stationary and known in this analysis. We discover that changes in *R_t_* alone can initiate inversions in the relative growth rate of different variants or epidemics. As far as we can tell, this phenomenon has not been explicitly investigated. Given that interventions can change *R_t_* in isolation or in combination with $w_{1}^{m}$ [15,18], this effect has the potential to be widespread. We determine the mathematical conditions for this inversion in the electronic supplementary material information.

Interestingly, the angular reproduction numbers of figure 4 do preserve an ordering that is consistent with growth rates, while maintaining the interpretability (e.g. threshold properties) of reproduction numbers. Hence, we argue that Ω*_t_* blends advantages from both *R_t_* and *r_t_* [4] and serves as a useful outbreak analytic for understanding and conveying the relative risk of spread of differing pathogens or pathogen strains, or of spread among different spatial and demographic groups. Recent studies have only begun to disentangle component drivers of transmission, including the differing effects that interventions can introduce (e.g. by defining the strength and speed of control measures [34]) and the diverse properties of antigenic variants [17]. We believe that *Ω* can play a distinctive role in accelerating these investigations.

### (e) Reproduction numbers for explanation or prediction?

We highlight an important but underappreciated subtlety when inferring the transmissibility of epidemics—that the value of accurately estimating *R*, *r* and *Ω* largely depends on if our aim is to explain or predict [35] the dynamics of epidemics. The above analyses have focused on characterizing transmissibility to explain mechanisms of spread and design interventions. For these problems, misestimation of parameters, such as *R*, can bias our assessment of outbreak risk and hence misinform the implementation of control measures. An important concurrent problem aims to predict the likely incidence of new infections from these estimates. This involves projecting the epidemic dynamics forward in time to infer upcoming infection patterns.

**Figure 5.**
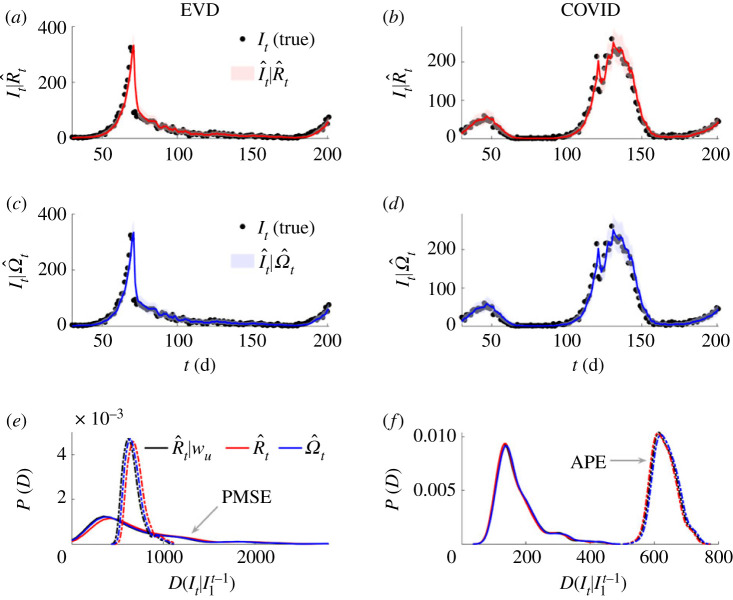
One-step-ahead prediction accuracy and model mismatch. We simulate 200 replicates of the epidemics from figure 3, which involve non-stationary changes to EVD and COVID-19 generation times. We use estimates of effective, *R*, and angular, *Ω*, reproduction numbers to produce successive one-step-ahead predictions and assess their accuracy to the simulated (true) incidence. Panels (*a–d*) provide a representative example of a single simulated epidemic (true incidence shown as black dots) and the *R* and *Ω* one-step ahead predictions (red and blue respectively with 95% credible intervals). In (*e,f*), we formally compute accuracy using distance metrics, *D*, based on accumulated prediction errors (APE, dashed) and prediction mean square errors (PMSE, solid) for all 200 replicates from *R*, *Ω* and *R* given knowledge of the generation time changes, i.e. *R*|*w*. We obtain distributions of *D* by applying kernel smoothing. We find negligible differences in predictive power from all approaches.

Here, we present evidence that the solution of this problem, at least over short projection time horizons, is robust to misspecification of generation times provided both the incorrect estimate and the misspecified denominator are used in conjunction. We repeat the analyses of figure 3 for 200 replicate epidemics and apply EpiFilter [25] to obtain the one-step-ahead predictive distributions $P(I_{t}|\;I_{1}^{t-1})$ for every *t*. We compute the predicted mean square error (PMSE) and the accumulated predictive error (APE). These scores, which we denote as $D(I_{t}|\;I_{1}^{t-1})$, average square errors between mean predictions and true incidence and sum log probabilities of observing the true incidence from the predicted distribution, respectively [36,37]. We plot the distributions of scores over replicates and illustrate individual predictions in figure 5.

We find only negligible differences among the one-step-ahead predictive accuracies of the *R* estimated given knowledge of the changing generation times (*R|w*), the *R* estimated assuming an unchanged (and hence wrongly specified) *w* and our inferred *Ω*. As APE and PMSE also measure model suitability, their similarity across the three estimates demonstrate that, if the problem of prediction is of interest, then incorrect generation time choices are not important as long as the erroneous denominator (Λ*_t_*) and estimate (*R_t_*) are used together. If this estimate is however used outside of the context of its denominator (e.g. if it is simply input into other studies), then inaccurate projections will occur (in addition to poor estimates). As multi-step-ahead predictions can be composed from iterated one-step-ahead ones [38], we conjecture that subtleties between prediction and explanation are likely to also apply on longer horizons.

### (f) Empirical example: COVID-19 in mainland China

**Figure 6.**
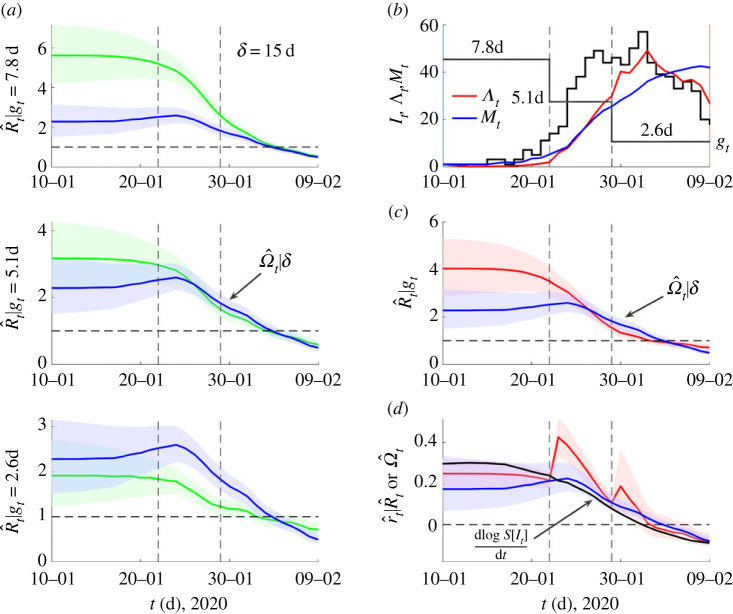
COVID-19 transmissibility in China under non-stationary generation times. We analyse COVID-19 data from [15], which spans 9 January 2020 to 13 February 2020 and is known to feature a serial interval distribution that shortened in mean substantially from 7.8 d to 2.6 d (change times are shown as grey vertical lines). We assume that the serial interval approximates the generation time well and replicate the analysis from fig. 2 of [15]. In (*a*), we compare estimates (green) of effective reproduction numbers, *R*, using fixed generation time distributions inferred in [15] (specified by their means *g*) against those of our angular reproduction number *Ω* (blue). We use EpiFilter [25] to obtain all estimates (means shown with 95% credible intervals) and find relative trends similar to those in fig. 2 of [15]. In (*b*), we plot the incidence (black) and the denominators we use to compute an *R* that does account for the generation time changes (*Λ*, red) and for *Ω* (M, blue). This *R* uses the different distributions inferred at the grey vertical change times (their means are in (*b*) and are also the fixed distributions of (*a*) in sequence). We plot these *R* and *Ω* estimates in (*c*). In (*d*), we show the growth rates that are inferred from the *R* and *Ω* estimates of (*c*) (red and blue, respectively) against that obtained from taking the smoothed log derivative (black).

We complete our analysis by illustrating the practical usability of *Ω* on an empirical case study where generation time changes are known to have occurred. In [15]*,* the dynamics of COVID-19 in mainland China are tracked across January and February 2020. Transmission pair data indicated that the serial interval of COVID-19 shortened across this period leading to biases in the inferred *R* if updated serial intervals are not used. Here serial intervals, which measure the lag between the symptom onset times of an infector and infectee, are used as a proxy for the generation time. Figure 6 presents our main results. We find *Ω* (blue), which requires no serial interval information, behaves similarly to the *R* (red) inferred from the time-changing *w*. Both metrics appear less biased than estimates of *R* (green) that assume a fixed serial interval. This is largely consistent with the original investigation in [15].

We see that *Ω* provides a lower assessment of the initial transmissibility as compared to the *R* that is best informed by the changing *w* but that both agree in general and in particular at the important threshold between super- and subcritical spread. Interestingly, *Ω* indicates no sharp changes at the *w* change-times. This follows because the incidence is too small for those changes to shape overall transmissibility and matches the gradual *w* changes originally inferred in [15]. The distributions used in figure 6 provide a piecewise approximation to these variations. We also compare *r* estimates derived from *R* (red, from [14]), *Ω* (blue, from equation (2.5)) and the empirical log gradient of smoothed incidence (black, $d\log S[I_{t}]/dt$ [4]). We find that the *r* from *Ω* agrees more closely with the empirical growth rate than the *r* from *R*, which somewhat by design shows jumps at the *w* change-points. While this analysis is not meant as a detailed study of COVID-19 in China, it does demonstrate the practical usefulness of *Ω*.

## 3. Discussion

Quantifying the time-varying transmissibility of a pathogen remains an enduring challenge in infectious disease epidemiology. Changes in transmissibility may signify shifts in the dynamics of an epidemic of relevance to both preparedness and policymaking. While this challenge has been longstanding, the statistics that we use to summarize transmissibility have evolved from dispersibility [39] and incidence to prevalence ratios [40] to cohort [41] and instantaneous [22] reproduction numbers. While the last, which we have denoted *R*, has become the predominant metric of transmissibility, all of these proposed statistics ultimately involve a ratio between new infections and a measure of active infections (i.e. the denominator). Deciding on appropriate denominators necessitates some notion (implicit or explicit) of a generation time [42].

Difficulties in characterizing these generation times and their changes substantially bias [6] estimates of transmissibility and have motivated recent works to propose the instantaneous growth rate, *r*, as a more reliable approach for inferring pathogen spread [20]. However, on its own, *r* is insufficient to resolve many of the transmission questions that *R* can answer and its computation may employ smoothing assumptions that are in some instances equivalent to the generation time ones behind *R* [4]. We formulated the novel angular reproduction number *Ω*, to merge some advantages from both *R* and *r* and to contribute to a more comprehensive view of transmissibility. By applying basic vector algebra (equations (2.1)–(2.3)), we encoded both changes to *R* and the generation time distribution, *w*, into a single time-varying metric, deriving *Ω*.

We found that *Ω* maintains the threshold properties and individual-level interpretability of *R* but responds to variations in *w*, in a manner consistent with *r* (figure 2). Moreover, *Ω* indicates variations in transmissibility caused by *R* and *w* without requiring measurement of generation times (figure 3). This is a consequence of its denominator, which is the root mean square of infections over a user-specified window *δ* that is relatively simple to tune (see Methods). We can interpret *Ω* = *a* > 1 as indicating that infections across *δ* need to be reduced by *a*^−1^. This reduces mean and root mean square infections by *a*^−1^ and causes *Ω* to equal 1. Further, *Ω* circumvents identifiability issues surrounding the joint inference or *R* and *w* [43] by refocusing on estimating the net changes produced by both. This improves our ability to *explain* the shifts in transmissibility underpinning observed epidemic dynamics and means *Ω* is essentially a reproduction number that provides individual-level interpretation of growth rates (equations (2.4)–(2.5)).

The benefits of this *r*–*Ω* correspondence are twofold. First, as interventions may alter *R*, *w* or *R* and *w* concurrently [15,18] situations can arise where *r* and *R* disagree across time on both the drivers and magnitude of transmissibility. While it may seem possible to minimize this issue by constructing alternative threshold statistics by directly combining *r* with assumed generation time structures, we find these statistics often exhibit worse performance and larger bias than *Ω* (electronic supplementary material information). Second, this disagreement can also occur when comparing pathogenic variants or epidemics (e.g. from diverse spatial or sociodemographic groups) with different but known and unchanging *w*. This study appears to be among the earliest to highlight these discrepancies, which can occur in multiple settings (see electronic supplementary material information). Realistic transmission landscapes possess all of the above complexities, meaning that relying solely on conventional measures of relative transmissibility can lead to contradictions.

We found that *Ω* consistently orders epidemics by growth rate while capturing notions of the average new infections per past infection (figure 4). This suggests *Ω* blends advantages from *R* and *r*, with clearer assumptions (choice of window *δ*). However, *Ω* offers no advantage if we want to *predict* epidemic dynamics (see [35] for more on prediction–explanation distinctions). For this problem, even an *R* inferred using a misspecified denominator performs equally well (figure 5). This follows as only the product of any reproduction number and its denominator matter when determining the next incidence value. Iterations of this product underpin multi-step-ahead predictions [38]. This may explain why autoregressive models, which ignore some characteristics of *w*, can serve as useful predictive models [44]. Other instances where *Ω* will not improve analysis are at times earlier than *δ* (due to edge effects [9]) and in periods of near zero incidence (there is no information to infer *R* either [24]). We summarized and compared key properties of *R*, *r* and *Ω* in table 1.

There are several limitations to our study. First, we only examined biases inherent to *R* due to the difficulty of measuring the generation time accurately and across time. While this is a major limitation of existing transmissibility metrics [15], practical surveillance data are also subject to under-reporting and delays, which can severely diminish the quality of any transmissibility estimates [23,43,45]. While *Ω* ameliorates issues due to generation time mismatch, it is as susceptible as *R* and *r* to surveillance biases and corrective algorithms (e.g. deconvolution methods [46]) should be applied before inferring *Ω*. Second, our analysis depends on renewal and compartmental epidemic models [22]. These assume random mixing and cannot account for realistic contact patterns. Despite this key structural uncertainty, there is evidence that well-mixed and network models are comparable when estimating transmissibility [47].

Although the above limitations can, in some instances, reduce the added value of improving the statistics summarizing transmissibility, we believe that *Ω* will be of practical and theoretical benefit, offering complementary insights to *R* and *r* and forming part of a more comprehensive epidemic analytic toolkit. Its similarity in formulation to *R* means it is as easy to compute using existing software and therefore can be deployed on dashboards and updated in real time to improve situational awareness. Further, *Ω* improves comparison and communication of the relative risks of circulating variants or epidemics among diverse groups, avoiding *R*–*r* contradictions provided the known parameter, *δ*, is fixed. This supplements *R*, which is hard to contextualize [20] when *w* is misspecified or varying and hence compare across groups, as each group may have distinct and correspondingly poorly specified denominators. Last, *Ω* can help probe analytical questions about how changes in *R* and *w* interact because it presents a common framework for testing how variations in either influence overall transmissibility.

## 4. Methods

### (a) Inferring angular reproduction numbers across time

We outline how to estimate Ω*_t_* given a time series of incident infections $I_{1}^{T}$, with *T* defining the present or last available data timepoint, i.e. 1 ≤ *t* ≤ *T*. Because Ω*_t_* simply replaces the total infectiousness Λ*_t_*, used for computing *R_t_*, with the root mean square of the new infection time series (figure 1), *M_t_*, we can obtain Ω*_t_* from standard *R_t_* estimation packages with minor changes. This requires evaluating *M_t_* over some user-defined, backward sliding window of size *δ*. Under a Poisson (Pois) renewal model this follows as in equation (4.1) for timepoint *t*.

4.1 $$P(I_{t}|\;I_{1}^{t-1},\delta )\equiv \mathrm{Pois}(\Omega_{t}M_{t}),\;\;\;\;\;\;\;M_{t}={\left(\frac{1}{\delta }\overset{t-1}{\underset{u=t-\delta \;}{\sum }}I_{u}^{2}\right)}^{1/2}.\;$$

The choice of *δ* is mostly arbitrary but should be sufficiently long to capture most of the likely probability mass of the unknown generation time but not overly long since it induces an edge effect (similar to the windows in [9,37]). We found a suitable heuristic to be twice or thrice the initial expected mean generation time (*g*_0_). We can then input *M_t_* and *I_t_* into packages such as EpiEstim [9] or EpiFilter [25] to estimate Ω*_t_* with 95% credible intervals.

Due to the similarity between computing *R_t_* and Ω*_t_*, we only specify the latter but highlight that replacing *M_t_* with Λ*_t_* yields the expressions for evaluating any equivalent quantities from *R_t_*. The only difference relates to how the growth rates *r_t_* are computed. We estimate *r_t_* from *R_t_* by applying the generation time, w, based transformation from [14]. For a correctly specified w this gives the same result as the smoothed derivative of the incidence curve [4]. We derive *r_t_* from Ω*_t_* using equation (2.5), which follows from rearranging equation (2.4) into $(2\delta r_{t}-\Omega_{t}^{2})e^{2\delta r_{t}-\Omega_{t}^{2}}=-\Omega_{t}^{2}e^{-\Omega_{t}^{2}}$. This expression then admits Lambert W function solutions. In all estimates of *r_t_*, we propagate uncertainty from the posterior distributions (see below) over *R_t_* or Ω*_t_*.

We applied EpiFilter in this study due to its improved extraction of information from $I_{1}^{T}$. This method assumes a random walk state model for our transmissibility metric as in equation (4.2) with ɛ*_t_*_−1_ as a normally distributed (Norm) noise term and η as a free parameter (default 0.1).

4.2 $$\Omega_{t}=\Omega_{t-1}+\left(\eta \sqrt{\Omega_{t-1}}\right)\epsilon_{t-1},\;P(\epsilon_{t-1})\equiv \mathrm{Norm}(0,\;1).$$

The EpiFilter approach uses Bayesian smoothing algorithms incorporating the models of equation (4.1)–(4.2) and outputs the complete posterior distribution $P(\Omega_{t}|\;I_{1}^{T},\delta )$ with *T* as the complete length of all available data (i.e. 1 ≤ *t* ≤ *T*). We compute our mean estimates ${\hat{\Omega }}_{t}$ and 95% credible intervals from this posterior distribution and these underlie our plots in figures 3 and 4.

EpiFilter also outputs the one-step-ahead predictive distributions $P(I_{t}|\;I_{1}^{t-1},\delta )$, which we use in figure 5. There we quantify predictive accuracy using the PMSE and the accumulated prediction error APE, defined as in equation (4.3) [36,37] with ${\hat{I}}_{t}$ as the posterior mean estimate from $P(I_{t}|\;I_{1}^{t-1},\delta )$ and $I_{t}^{*}$ as the true simulated incidence. These are computed with $P(\Omega_{t-1}|\;I_{1}^{t-1},\delta )$ and not $P(\Omega_{t}|\;I_{1}^{T},\delta )$, ensuring no future information is used.

4.3 $$\begin{array}{rl} & \mathrm{PMSE}=\frac{1}{T-\delta }\overset{T}{\underset{t=\delta +1\;}{\sum }}{\left(I_{t}^{*}-{\hat{I}}_{t}\right)}^{2},\\ & \;\mathrm{APE}=\overset{T}{\underset{t=\delta +1\;}{\sum }}-\log P(I_{t}=I_{t}^{*}|\;I_{1}^{t-1},\delta ). \end{array}$$

We collectively refer to these as distance metrics $D(I_{t}|\;I_{1}^{t-1})$ and construct their distributions, P(D), over many replicates of simulated epidemics. Last, we use $P(\Omega_{t}|\;I_{1}^{T},\delta )$ to compute the posterior distribution of the growth rate $P(r_{t}|\;I_{1}^{T},\delta )$ and hence its estimates as in equation (2.5). More details on the EpiFilter algorithms are available elsewhere [25,31,48]. We supply open-source code to reproduce all analyses at https://github.com/kpzoo/Omega as well as functions in Matlab and R to allow users to estimate Ω*_t_* from their own data.

## Data Availability

All data and code underlying the analyses and figures of this work are freely available (in R and Matlab) at: https://github.com/kpzoo/Omega. Supplementary material is available online [49].

## References

[1] Anderson R, Donnelly C, Hollingsworth D, Keeling M, Vegvari C, Baggaley R. 2020 Reproduction number (*R*) and growth rate (*r*) of the COVID-19 epidemic in the UK: methods of estimation, data sources, causes of heterogeneity. Royal Society technical report. See https://royalsociety.org/-/media/policy/projects/set-c/set-covid-19-R-estimates.pdf.

[2] Li Y, Campbell H, Kulkarni D, Harpur A, Nundy M, Wang X, Nair H. 2021 The temporal association of introducing and lifting non-pharmaceutical interventions with the time-varying reproduction number (R) of SARS-CoV-2: a modelling study across 131 countries. Lancet Infect. Dis. **21**, 193-202. (10.1016/S1473-3099(20)30785-4) 33729915 PMC7581351

[3] Volz E et al. 2021 Assessing transmissibility of SARS-CoV-2 lineage B.1.1.7 in England. Nature **593**, 266-269. (10.1038/s41586-021-03470-x) 33767447

[4] Parag KV, Thompson RN, Donnelly CA. 2022 Are epidemic growth rates more informative than reproduction numbers? J. R. Stat. Soc. A **185**, S5-S15. (10.1111/rssa.12867) PMC9347870 35942192

[5] Svensson A. 2007 A note on generation times in epidemic models. Math. Biosci. **208**, 300-311. (10.1016/j.mbs.2006.10.010) 17174352

[6] Britton T, Scalia Tomba G. 2019 Estimation in emerging epidemics: biases and remedies. J. R. Soc. Interface **16**, 20180670. (10.1098/rsif.2018.0670) 30958162 PMC6364646

[7] Champredon D, Dushoff J. 2015 Intrinsic and realized generation intervals in infectious-disease transmission. Proc. Biol. Sci. **282**, 20152026. (10.1098/rspb.2015.2026) 26674948 PMC4707754

[8] Nishiura H. 2010 Time variations in the generation time of an infectious disease: implications for sampling to appropriately quantify transmission potential. Math. Biosci. Eng. **7**, 851-869. (10.3934/mbe.2010.7.851) 21077712

[9] Cori A, Ferguson NM, Fraser C, Cauchemez S. 2013 A new framework and software to estimate time-varying reproduction numbers during epidemics. Am. J. Epidemiol. **178**, 1505-1512. (10.1093/aje/kwt133) 24043437 PMC3816335

[10] Ganyani T, Kremer C, Chen D, Torneri A, Faes C, Wallinga J, Hens N. 2020 Estimating the generation interval for coronavirus disease (COVID-19) based on symptom onset data, March 2020. Eurosurveillance **25**, 2000257. (10.2807/1560-7917.ES.2020.25.17.2000257) 32372755 PMC7201952

[11] Hethcote HW. 2000 The mathematics of infectious diseases. SIAM Rev. **42**, 599-653. (10.1137/S0036144500371907)

[12] Parag KV. 2021 Sub-spreading events limit the reliable elimination of heterogeneous epidemics. J. R. Soc. Interface **18**, 20210444. (10.1098/rsif.2021.0444) 34404230 PMC8371363

[13] Anderson R, May R. 1991 Infectious diseases of humans: dynamics and control. Oxford, UK: Oxford University Press.

[14] Wallinga J, Lipsitch M. 2007 How generation intervals shape the relationship between growth rates and reproductive numbers. Proc. R. Soc. B **274**, 599-604. (10.1098/rspb.2006.3754) PMC1766383 17476782

[15] Ali ST, Wang L, Lau EHY, Xu X-K, Du Z, Wu Y, Leung GM, Cowling BJ. 2020 Serial interval of SARS-CoV-2 was shortened over time by nonpharmaceutical interventions. Science **369**, 1106-1109. (10.1126/science.abc9004) 32694200 PMC7402628

[16] Kenah E, Lipsitch M, Robins JM. 2008 Generation interval contraction and epidemic data analysis. Math. Biosci. **213**, 71-79. (10.1016/j.mbs.2008.02.007) 18394654 PMC2365921

[17] Hart WS, Miller E, Andrews NJ, Waight P, Maini PK, Funk S, Thompson RN. 2022 Generation time of the alpha and delta SARS-CoV-2 variants: an epidemiological analysis. Lancet Infect. Dis. **22**, 603-610. (10.1016/S1473-3099(22)00001-9) 35176230 PMC8843191

[18] Favero M, Scalia Tomba G, Britton T. 2022 Modelling preventive measures and their effect on generation times in emerging epidemics. J. R. Soc. Interface **19**, 20220128. (10.1098/rsif.2022.0128) 35702865 PMC9198515

[19] Kraemer MUG, Pybus OG, Fraser C, Cauchemez S, Rambaut A, Cowling BJ. 2021 Monitoring key epidemiological parameters of SARS-CoV-2 transmission. Nat. Med. **27**, 1854-1855. (10.1038/s41591-021-01545-w) 34750555

[20] Pellis L et al. 2021 Challenges in control of COVID-19: short doubling time and long delay to effect of interventions. Phil. Trans. R. Soc. B **376**, 20200264. (10.1098/rstb.2020.0264) 34053267 PMC8165602

[21] UK Government. 2021 The R value and growth rate. See https://www.gov.uk/guidance/the-r-value-and-growth-rate.

[22] Fraser C. 2007 Estimating individual and household reproduction numbers in an emerging epidemic. PLoS ONE **2**, e758. (10.1371/journal.pone.0000758) 17712406 PMC1950082

[23] Parag KV, Donnelly CA, Zarebski AE. 2022 Quantifying the information in noisy epidemic curves. Nat. Comput. Sci. **2**, 584-594. (10.1038/s43588-022-00313-1) 38177483

[24] Parag KV, Donnelly CA. 2022 Fundamental limits on inferring epidemic resurgence in real time using effective reproduction numbers. PLoS Comput. Biol. **18**, e1010004. (10.1371/journal.pcbi.1010004) 35404936 PMC9022826

[25] Parag KV. 2021 Improved estimation of time-varying reproduction numbers at low case incidence and between epidemic waves. PLoS Comput. Biol. **17**, e1009347. (10.1371/journal.pcbi.1009347) 34492011 PMC8448340

[26] Torneri A, Libin P, Scalia Tomba G, Faes C, Wood JG, Hens N. 2021 On realized serial and generation intervals given control measures: the COVID-19 pandemic case. PLoS Comput. Biol. **17**, e1008892. (10.1371/journal.pcbi.1008892) 33780436 PMC8031880

[27] Lloyd-Smith JO, Schreiber SJ, Kopp PE, Getz WM. 2005 Superspreading and the effect of individual variation on disease emergence. Nature **438**, 355-359. (10.1038/nature04153) 16292310 PMC7094981

[28] Bettencourt LMA, Ribeiro RM. 2008 Real time bayesian estimation of the epidemic potential of emerging infectious diseases. PLoS ONE **3**, e2185. (10.1371/journal.pone.0002185) 18478118 PMC2366072

[29] Gostic KM et al. 2020 Practical considerations for measuring the effective reproductive number, Rt. PLoS Comput. Biol. **16**, e1008409. (10.1371/journal.pcbi.1008409) 33301457 PMC7728287

[30] Lehtonen J. 2016 The Lambert W function in ecological and evolutionary models. Methods Ecol. Evol. **7**, 1110-1118. (10.1111/2041-210X.12568)

[31] Parag KV, Cowling BJ, Donnelly CA. 2021 Deciphering early-warning signals of SARS-CoV-2 elimination and resurgence from limited data at multiple scales. J. R. Soc. Interface **18**, 20210569. (10.1098/rsif.2021.0569) 34905965 PMC8672070

[32] Van Kerkhove MD, Bento AI, Mills HL, Ferguson NM, Donnelly CA. 2015 A review of epidemiological parameters from Ebola outbreaks to inform early public health decision-making. Sci. Data **2**, 150019. (10.1038/sdata.2015.19) 26029377 PMC4443880

[33] Ferguson N et al. 2020 Impact of non-pharmaceutical interventions (NPIs) to reduce COVID-19 mortality and healthcare demand. London, UK: Imperial College London. (10.25561/77482) PMC7140590 32270376

[34] Dushoff J, Park SW. 2021 Speed and strength of an epidemic intervention. Proc. Biol. Sci. **288**, 20201556. (10.1098/rspb.2020.1556) 33757359 PMC8059560

[35] Shmueli G. 2010 To explain or to predict? Stat. Sci. **25**, 289-310. (10.1214/10-STS330)

[36] Wagenmakers E-J, Grünwald P, Steyvers M. 2006 Accumulative prediction error and the selection of time series models. J. Math. Psychol. **50**, 149-166. (10.1016/j.jmp.2006.01.004)

[37] Parag KV, Donnelly CA. 2020 Using information theory to optimise epidemic models for real-time prediction and estimation. PLoS Comput. Biol. **16**, e1007990. (10.1371/journal.pcbi.1007990) 32609732 PMC7360089

[38] Marcellino M, Stock JH, Watson MW. 2006 A comparison of direct and iterated multistep AR methods for forecasting macroeconomic time series. J. Econ. **135**, 499-526. (10.1016/j.jeconom.2005.07.020)

[39] Nishiura H, Chowell G, Heesterbeek H, Wallinga J. 2010 The ideal reporting interval for an epidemic to objectively interpret the epidemiological time course. J. R. Soc. Interface **7**, 297-307. (10.1098/rsif.2009.0153) 19570792 PMC2842610

[40] White PJ, Ward H, Garnett GP. 2006 Is HIV out of control in the UK? An example of analysing patterns of HIV spreading using incidence-to-prevalence ratios. AIDS **20**, 1898-1901. (10.1097/01.aids.0000244213.23574.fa) 16954735

[41] Wallinga J, Teunis P. 2004 Different epidemic curves for severe acute respiratory syndrome reveal similar impacts of control measures. Am. J. Epidemiol. **160**, 509-516. (10.1093/aje/kwh255) 15353409 PMC7110200

[42] Yan P. 2008 Separate roles of the latent and infectious periods in shaping the relation between the basic reproduction number and the intrinsic growth rate of infectious disease outbreaks. J. Theor. Biol. **251**, 238-252. (10.1016/j.jtbi.2007.11.027) 18191153

[43] Azmon A, Faes C, Hens N. 2014 On the estimation of the reproduction number based on misreported epidemic data. Stat. Med. **33**, 1176-1192. (10.1002/sim.6015) 24122943

[44] Bracher J, Held L. 2020 Endemic-epidemic models with discrete-time serial interval distributions for infectious disease prediction. Int. J. Forecast. **38**, 1221-1233. (10.1016/j.ijforecast.2020.07.002)

[45] Dalziel BD, Lau MSY, Tiffany A, McClelland A, Zelner J, Bliss JR, Grenfell BT. 2018 Unreported cases in the 2014–2016 Ebola epidemic: spatiotemporal variation, and implications for estimating transmission. PLoS Negl. Trop. Dis. **12**, e0006161. (10.1371/journal.pntd.0006161) 29357363 PMC5806896

[46] Goldstein E, Dushoff J, Ma J, Plotkin JB, Earn DJD, Lipsitch M. 2009 Reconstructing influenza incidence by deconvolution of daily mortality time series. Proc. Natl Acad. Sci. USA **106**, 21 825-21 829. (10.1073/pnas.0902958106) PMC2796142 20080801

[47] Liu Q-H, Ajelli M, Aleta A, Merler S, Moreno Y, Vespignani A. 2018 Measurability of the epidemic reproduction number in data-driven contact networks. Proc. Natl Acad. Sci. USA **115**, 12 680-12 685. (10.1073/pnas.1811115115) PMC6294899 30463945

[48] Sarrka S. 2013 Bayesian filtering and smoothing. Cambridge, UK: Cambridge University Press.

[49] Parag KV, Cowling BJ, Lambert BC. 2023 Angular reproduction numbers improve estimates of transmissibility when disease generation times are misspecified or time-varying. Figshare. (10.6084/m9.figshare.c.6837175) PMC10523088 37752839

